# Characterizing synaptic protein development in human visual cortex enables alignment of synaptic age with rat visual cortex

**DOI:** 10.3389/fncir.2015.00003

**Published:** 2015-02-12

**Authors:** Joshua G. A. Pinto, David G. Jones, C. Kate Williams, Kathryn M. Murphy

**Affiliations:** ^1^McMaster Integrative Neuroscience Discovery and Study (MiNDS) Program, McMaster UniversityHamilton, ON, Canada; ^2^Pairwise Affinity Inc.Dundas, ON, Canada; ^3^Psychology, Neuroscience and Behavior, McMaster UniversityHamilton, ON, Canada

**Keywords:** human cortex, synaptic proteins, development, rat cortex, visual cortex

## Abstract

Although many potential neuroplasticity based therapies have been developed in the lab, few have translated into established clinical treatments for human neurologic or neuropsychiatric diseases. Animal models, especially of the visual system, have shaped our understanding of neuroplasticity by characterizing the mechanisms that promote neural changes and defining timing of the sensitive period. The lack of knowledge about development of synaptic plasticity mechanisms in human cortex, and about alignment of synaptic age between animals and humans, has limited translation of neuroplasticity therapies. In this study, we quantified expression of a set of highly conserved pre- and post-synaptic proteins (Synapsin, Synaptophysin, PSD-95, Gephyrin) and found that synaptic development in human primary visual cortex (V1) continues into late childhood. Indeed, this is many years longer than suggested by neuroanatomical studies and points to a prolonged sensitive period for plasticity in human sensory cortex. In addition, during childhood we found waves of inter-individual variability that are different for the four proteins and include a stage during early development (<1 year) when only Gephyrin has high inter-individual variability. We also found that pre- and post-synaptic protein balances develop quickly, suggesting that maturation of certain synaptic functions happens within the 1 year or 2 of life. A multidimensional analysis (principle component analysis) showed that most of the variance was captured by the sum of the four synaptic proteins. We used that sum to compare development of human and rat visual cortex and identified a simple linear equation that provides robust alignment of synaptic age between humans and rats. Alignment of synaptic ages is important for age-appropriate targeting and effective translation of neuroplasticity therapies from the lab to the clinic.

## Introduction

Neuroplasticity is tightly regulated by synaptic mechanisms that promote or limit changes in neural circuits. These mechanisms ultimately determine how we see, hear, feel, learn, and think. Studies from many areas of neuroscience have shown that the brain adapts to new demands and that adaptation happens most easily during development. Plasticity during the sensitive period is usually adaptive and supports long-term maturation of important perceptual and cognitive skills (Banks et al., 1975; Stiles, 2000); but it can also be maladaptive, such as when abnormal visual experience causes poor visual perception known as amblyopia (lazy-eye) (Wiesel and Hubel, 1965). The synaptic mechanisms regulating plasticity in the cortex are best understood for sensory areas. Tapping into adaptive neuroplasticity could improve therapies for conditions such as brain injury, neuropsychiatric disorders, neurodevelopmental disorders, and neurodegeneration. An NIH report about neuroplasticity research, however, noted that few of advances have translated from the lab into established clinical interventions (Cramer et al., 2011). As with all therapies, successful translation hinges on a good fit of the animal model with the human condition, which for neuroplasticity, relies on the ability to align developmental stages between animals and humans.

There are many benefits of a comparative approach to discovering features of synapse development that generalize across species and identify periods of heightened plasticity. For example, aligning brain age between animal models and humans would help unify our knowledge of brain development from animal studies using modern neurobiological techniques to human brain imaging with novel methods (e.g., imaging cortical myelin). Bridging that gap has been limited by two important factors: the pace of development differs among species; and the capacity for plasticity changes across the lifespan.

For human cortical development, the traditional view is that primary sensory areas in the cortex (e.g., primary visual and auditory cortex) have an early sensitive period that peaks within the first year or two of life while higher-order cortical areas have much longer sensitive periods extending through childhood and even into adolescence. That view is strongly influenced by classic neuroanatomical studies that counted synapses in postmortem tissue and found a rapid postnatal rise then fall in the number of synapses in primary visual and auditory cortex but slower changes in higher-order cortical areas (Huttenlocher et al., 1982; Huttenlocher and de Courten, 1987; Huttenlocher and Dabholkar, 1997). Those synapse counts have been used to suggest that primary visual cortex (V1) mediates an early sensitive period ending in infancy while more prolonged sensitive periods are mediated by high-order cortical areas (Lewis and Maurer, 2005). More recent studies of human visual cortex development using MRI to assess cortical thickness (Gogtay et al., 2004; Sowell et al., 2004; Shaw et al., 2008) or Western blotting to quantify neurotransmitter proteins (Murphy et al., 2005; Pinto et al., 2010; Williams et al., 2010) have reopened the debate of how quickly V1 matures. These newer studies found gradual development of V1 that continues well into late childhood. Perhaps slow development of V1 contributes to prolonged maturation of visual perception (Gervan et al., 2011), and a long sensitive period for amblyopia (Epelbaum et al., 1993; Keech and Kutschke, 1995; Lewis and Maurer, 2005). Despite this accumulating evidence, the field has been slow to update the model for synaptic development of human V1, which makes it challenging to align neurodevelopmental stages between humans and animal models.

Typically, developmental stages among species are aligned using the reproductive lifespan. The pace of sexual maturation, however, is very different among species, particularly for rodents that are precocious, and humans, that progress more slowly through similar stages of sexual maturation. Slower development may also lead to greater inter-individual variability and more opportunity for variations in experience to affect human brain development. Thus, it is problematic to use reproductive stages to align brain age between species.

To address this inter-species brain age alignment problem, we developed a synapse-based approach, similar to the simple method for rapid and reliable quantification of neuron number in human cortex (Herculano-Houzel, 2012). Our approach uses Western blotting and a small set of highly conserved synaptic proteins (Synapsin, Synaptophysin, PSD-95, Gephyrin) to measure changes in the cortex across the lifespan. The four synaptic proteins serve key pre- and post-synaptic functions linked to development and the capacity for neuroplasticity. The maturation of sensory evoked firing patterns in cortical neurons is essential for experience-dependent plasticity and depends on pre-synaptic development of neurotransmitter release. Synapsin and Synaptophysin regulate exo- (Bähler et al., 1990) and endo-cytosis (Kwon and Chapman, 2011) of neurotransmitter vesicles, respectively, and are therefore necessary for transmitter release (Hopf et al., 2002). The balance between excitation and inhibition (E-I balance) is another key mechanism for triggering and regulating sensitive period plasticity (Hensch and Fagiolini, 2005). An abrupt post-synaptic shift in relative amounts of excitatory and inhibitory receptors is tied to the start of the sensitive period in visual cortex. To capture the shift in E-I balance, we quantify the excitatory and inhibitory receptor scaffolding proteins PSD-95 and Gephyrin, respectively. Interactions between PSD-95 and Gephyrin regulate the number of excitatory and inhibitory synapses and affect the physiological E-I balance (Prange et al., 2004; Lardi-Studler et al., 2007; Keith and El-Husseini, 2008).

Here we apply our synapse-based approach to determine the pace of development for human visual cortex, inter-individual variability, and the alignment of cortical age between humans and rats. First, we quantify expression of four highly conserved synaptic proteins in postmortem human visual cortex tissue samples covering a wide range of ages. Then, we use multidimensional analysis (principal components) to characterize the developmental trajectory and inter-individual variability (Fano factor) in synaptic development. Finally, we use model fitting to determine the equations that capture synaptic development in human visual cortex, and apply a simple transformation to align synaptic development between rats and humans.

## Methods

### Samples and tissue

Tissue samples were obtained from the Brain and Tissue Bank for Developmental Disorders at the University of Maryland (Baltimore, MD) and use of the tissue was approved by McMaster University research ethics board. The samples were from the posterior pole of the left hemisphere of human visual cortex, including both superior and inferior portions of the calcarine fissure. A small piece was cut from central visual field representation of V1 according to the gyral and sulcal landmarks. The samples were from 26 individuals ranging in age from 20 days to 80 years (Table 1). All samples were obtained within 23 h postmortem, and at the Brain and Tissue Bank were fresh frozen after being sectioned coronally in 1 cm intervals, rinsed with water, blotted dry, placed in a quick-freeze bath (dry ice and isopentane), and stored frozen (−70°C). The individuals had no history of neurological or mental health disorders.

**Table 1:** **Human tissue samples**.

Human age	Postmortem interval (h)	Sex	Cause of death
20 days	14	F	Pneumonia
86 days	23	F	Not known
96 days	12	M	Bronchopneumonia
98 days	16	M	Cardiovascular disorder
119 days	22	M	Bronchopneumonia
120 days	23	M	Pneumonia
133 days	16	M	Accidental
136 days	11	F	Pneumonia
273 days	10	M	Sudden infant death syndrome
1.34 years	21	M	Dehydration
2.16 years	21	F	Cardiovascular disorder
3.34 years	11	F	Accidental
4.56 years	15	M	Accidental
5.39 years	17	M	Accidental
8.59 years	20	F	Surgical complications
12.45 years	22	M	Cardiovascular disorder
13.27 years	5	M	Asphyxia
15.22 years	16	M	Multiple injuries
19.21 years	16	F	Multiple injuries
22.98 years	4	M	Multiple injuries
32.61 years	13	M	Cardiovascular disorder
50.43 years	8	M	Cardiovascular disorder
53.90 years	5	F	Cardiovascular disorder
69.30 years	12	M	Cardiovascular disorder
71.91 years	9	F	Multiple medical disorders
79.50 years	14	F	Drug overdose

*The age and postmortem interval for each of the human cortical tissue samples*.

### Tissue-sample preparation

To quantify the available pool of synaptic proteins, tissue samples (50–100 mg) were suspended in cold homogenization buffer (1 ml buffer:50 mg tissue, 0.5 mM DTT, 1 mM EDTA, 2 mM EGTA, 10 mM HEPES, 10 mg/L leupeptin, 100 nM microcystin, 0.1 mM PMSF, 50 mg/L soybean trypsin inhibitor) and homogenized in a glass-glass Dounce homogenizer (Kontes, Vineland, NJ). The homogenized sample was removed and added to 10% sodium-dodecyl-sulfate (SDS). Protein concentrations were determined using the bicinchonic acid (BCA) assay guidelines (Pierce, Rockford, IL). A control sample was made by combining a small amount of the prepared tissue sample from each of the 26 samples.

### Immunoblotting

The homogenate samples (25 μg) were separated on SDS polyacrylamide gels (4–20% SDS-PAGE; Thermo Scientific, Waltham, MA) and transferred to polyvinylidene difluoride (PVDF-FL) membranes (Millipore, Billerica, MA). We used whole homogenate samples for two reasons: first, the proteins we quantified have high abundances; second, PSD-95 and Gephyrin are motile among a localized group of synapses (Gray et al., 2006; Dobie and Craig, 2011). Each sample was run 2–4 times. Blots were pre-incubated in blocking buffer (Odyssey Blocking Buffer, Li-cor Biosciences; Lincoln, NE; 1:1 with PBS) for 1 h, then incubated in primary antibody overnight at 4°C using the following concentrations: GAPDH, 1:4000 (Imgenex, San Diego, CA); Synapsin 1, 1:8000 (Invitrogen, Carlsbad, CA); Synaptophysin, 1:2000 (Sigma-Aldrich, St. Louis, MO); PSD-95, 1:32000 (Millipore, Billerica, MA); Gephyrin, 1:2000 (Millipore, Billerica, MA). These antibodies were selected after verifying that we got comparable bands from multiple species. The blots were washed with PBS containing 0.05% Tween (PBS-T) (Sigma, St. Louis, MO) (3 × 10 min), incubated for 1 h at room temperature with the appropriate IRDye labeled secondary antibody, (Anti-Mouse, 1:8000, Anti-Rabbit, 1:10,000) (Li-cor Biosciences; Lincoln, NE), and washed in PBS-T (3 × 10 min). The bands were visualized using the Odyssey scanner (Li-cor Biosciences; Lincoln, NE). The combination of the IRDye secondary antibodies and Odyssey scanner system provides a wide linear dynamic range so that both strong and weak bands could be quantified on the same blot. We determined that both the amount of protein loaded in each well and the concentration of each antibody were within the linear range. The blots were stripped and prepared to be re-probed with additional antibodies (Blot Restore Membrane Rejuvenation kit, Chemicon International, Temecula, CA).

### Analysis

To analyze the bands, we scanned the blots (Odyssey Infrared Scanner) and quantified the bands using densitometry (Licor Odyssey Software version 3.0; Li-cor Biosciences; Lincoln, NE). The Odyssey system uses near infrared-dyes for antibody detection, providing 16–250 fold wider linear range than chemilu-minescence. Density profiles were determined by performing a subtraction of the background, integrating the pixel intensity across the area of the band, and dividing the intensity by the width of the band to control for variations in lane width. GAPDH was used as the loading control and for each sample the expression of the synaptic proteins was divided by GAPDH expression. A control sample (a mixture of all the samples) was run on all of the gels and for each blot the density of each sample was normalized relative to the control (Sample density/Control density). Finally, for each protein, expression levels across runs were normalized using the average expression of the synaptic protein.

The results were plotted in two ways to visualize the developmental trajectories of the proteins. First, changes in expression across the lifespan were plotted using scattergrams for each protein that included expression levels from all runs (light gray dots), as well as the average expression from each sample (black dots). To help quantify the patterns of change in expression, we used a model-fitting approach (Christopoulos and Lew, 2000) and determined the best curve-fit to the data (gray dots) using the online tool zunzun.com. The best fitting curve was found by least squares providing the goodness-of-fit (R) and statistical significance of the fit (p). Synapsin expression was well fit by a decay function (y = a + b * exp (−t/τ)), and the time constant (τ) was determined. Adult levels were defined as 3τ, a time when 87.5% of the change in protein expression had taken place. Synaptophysin was not well fit by any functions, so a simple descriptive weighted average was plotted. PSD-95 and Gephyrin expression were well fit by Gaussian functions (y = a + b * exp (−((t−peak)^2^)/c)) since expression of both increased then decreased. Second, to compare changes among developmental stages, samples were grouped by developmental stage (Neonates: <0.3 years; Infants: 0.3–1 years; Young Children: 1–4 years; Older Children: 5–11 years; Teens: 12–20 years; Young Adults: 21–55 years; Older Adults: >55 years) and histograms were plotted showing the mean expression level of the synaptic protein and the standard error of the mean for each age group. Statistical comparisons between groups were made using an analysis of variance and, when significant (*p* < 0.05), Tukey’s *post hoc* comparisons were done.

We examined changes in the inter-individual variability for the four synaptic proteins by calculating the Fano-Factor (Variance-to-Mean Ratio—VMR). At each age, we determined the mean and variance within a box that included the 2 adjacent ages. The VMRs were plotted as scatter plots and functions were fit to describe ages when there was higher or lower inter-individual variability. The best fitting curves for the VMRs were determined by least squares (Synaptophysin and PSD-95, peak function y = a * exp (b/x + c * x); Gephyrin, decay function y = a + (b − a)/(1 + (x/c)^d^)).

We quantified the relationship between pre- and post-synaptic proteins by calculating two indices that measured the developmental differences between the pair of pre-synaptic (Synapsin and Synaptophysin) or post-synaptic proteins (PSD-95 and Gephyrin). The indices provide an indication of synaptic development because each pair of proteins is functionally related: Synapsin and Synaptophysin expression is required for pre-synaptic function and stabilization of pre-synaptic boutons (Hopf et al., 2002); interactions between PSD-95 and Gephyrin regulate the number of excitatory and inhibitory synapses and affect the physiological E/I balance (Prange et al., 2004; Lardi-Studler et al., 2007; Keith and El-Husseini, 2008). In addition, this type of contrast index is a common approach in signal processing to determine the quality of the signal and here provided an analysis of pre- or post-synaptic development. Pre-Synaptic Index = [(Synapsin − Synaptophysin)/(Synapsin + Synaptophysin)], Post-Synaptic Index = [(PSD-95 − Gephyrin)/(PSD-95 + Gephyrin)]. The indices were plotted as described above, and exponential decay functions were fit to the scatterplots.

### Principal component analysis

A multivariate analysis of the expression pattern for all proteins in human visual cortex was done using principal component analysis (PCA) using procedures we developed for analyzing synaptic proteins in cat (Beston et al., 2010) and rat (Pinto et al., 2013) visual cortex. Protein expression was compiled into an mxn matrix. The m rows (4) represent the proteins (Synapsin, Synaptophysin, PSD-95, and Gephyrin), and the n columns (52) represent protein expression levels for 2 runs of the 26 samples. The data were centered by subtracting the mean column vector, and then a singular value decomposition (SVD) was applied to calculate the principal components in Matlab (The Mathworks, Inc., Natick, MA). The SVD represents the protein expression levels from one sample as a vector in high dimensional space. The PCA identifies the directions in “protein expression space” that capture the variance in all the data from the human visual cortex.

The analysis identified four principal components. A commonly used rule of thumb to determine how many components are significant is to include sequential principal components accounting for up to 80% of the cumulative variance. In addition, we analyzed the significance of the principle components using a bootstrapping method where we compared the principle components calculated from the experimental data with components calculated from a simulated data-set. The simulated data-set had 4 rows (proteins) and 26 columns (samples), and the simulated protein expression levels were drawn randomly from a normal distribution with the same mean and standard deviation as the experimental data. A Monte Carlo simulation was run with 100,000 iterations, a PCA was performed for each iteration to calculate how much of the residual variance was accounted for by each of the four principal components. Our experimental principal components were deemed to be statistically significant if they accounted for a much greater proportion of the residual variance than would be expected from random chance. For example, a principal component was significant with *p* < 0.05 if it accounted for more of the residual variance than was observed in 95% of the simulated iterations. Using the Monte Carlo simulation we found that only PCA 1 and 2 were significant.

To determine biological links and to aid interpretation of the significant principal components, we used an approach that we developed previously (Beston et al., 2010) and calculated correlations between each significant principal component and several biologically relevant measures. These included: expression levels of the four proteins, the pre- and post-synaptic index, and the sum of the four synaptic proteins (synaptic protein expression). The significance level for identifying potential biological correlates was adjusted to *p* < 0.0035 using the Bonferroni correction for multiple comparisons. The PCA results were visualized using scatterplots and histograms as described above, with PCA coordinates on the y-axis, and age on the x-axis. To describe the pattern of change in the PCA scatterplots, we fit peak or linear functions to the data.

## Results

### Postmortem interval and GAPDH expression

The tissue samples were collected over a range of postmortem intervals (4–23 h) and our first step was to determine whether there were significant correlations between postmortem interval and expression of the synaptic proteins. We found no significant (*p* > 0.05) correlations between expression levels and postmortem interval for any of the four proteins (GAPDH, *R* = 0.04; Synapsin, *R* = 0.16; Synaptophysin, *R* = 0.05; Gephyrin, *R* = 0.08; PSD95, *R* = 0.10).

**Figure 1:**
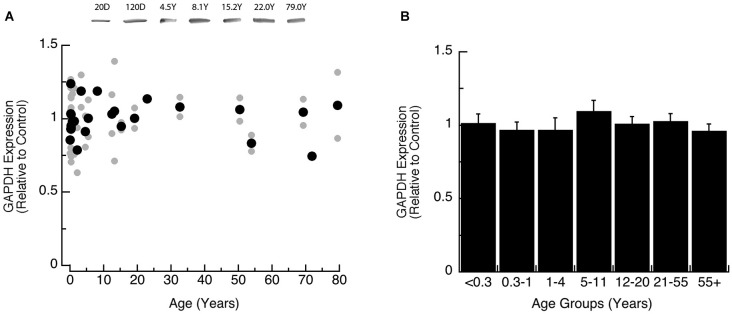
**Developmental changes in GAPDH expression in human visual cortex. (A)** Gray dots are results from all runs, and black dots are the average for each sample. Example bands are shown above the graph. **(B)** Group means and standard error for each developmental group.

We also analyzed expression of GAPDH, the loading control for this study, to determine if it changed across the lifespan. We found that the expression level of GAPDH was constant across the lifespan when analyzed by either model-fitting (Figure 1A, *R* = 0.05), or comparison among the age groups (Figure 1B; ANOVA, *p* = 0.87).

### Development of pre-synaptic vesicle cycling mechanisms

To examine pre-synaptic development in human visual cortex across the lifespan, we quantified the expression of two proteins, Synapsin and Synaptophysin. Both proteins are components of the synaptic vesicle membrane and involved in different aspects of synaptic vesicle cycling. Synapsin regulates the pool of synaptic vesicles available for exocytosis (Bähler et al., 1990), and Synaptophysin regulates the kinetics of synaptic vesicle endocytosis (Kwon and Chapman, 2011). In addition, Synapsin is the most specific marker for pre-synaptic terminals (Micheva et al., 2010). Together, expression levels of Synapsin and Synaptophysin provide information about function and number of pre-synaptic terminals.

**Figure 2:**
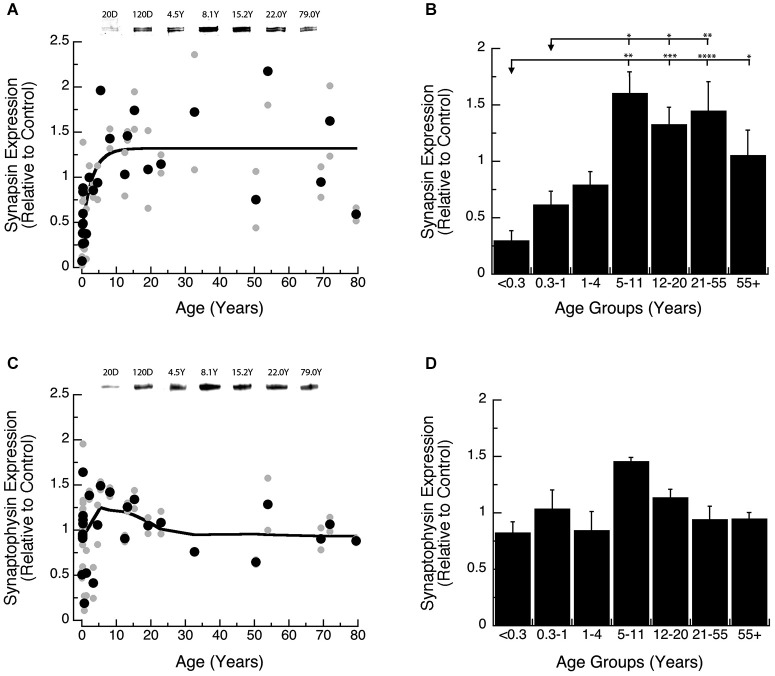
**Developmental changes in Synapsin (A,B) and Synaptophysin (C,D) expression in human visual cortex. (A,C)** Gray dots are results from all runs, and black dots are the average for each sample. Example bands are shown above the graphs. **(B,D)** Group means and standard error for each developmental stage are plotted. **(A)** An exponential decay function was fit to all the Synapsin data points (*R* = 0.66, *p* < 0.0001), and adult levels are defined as 3t (3t = 8.7 +/− 5.1 years). **(B)** There was a significant difference in expression of Synapsin between the groups (ANOVA, *p* < 0.0001), and the statistical significance of the difference between pairs of development stages as determined by Tukey’s *post hoc* comparisons are plotted (*p < 0.05; **p < 0.01; ***p < 0.001; ****p < 0.0001). **(C)** A weighted average fit was plotted to all of the Synaptophysin data points to describe pattern of change. **(D)** There was no significant difference in expression of Synaptophysin between the groups (ANOVA, *p* = 0.09).

We found a gradual increase in expression levels of Synapsin during development of human visual cortex, and the changes were well fit by a τ decay function (Figures 2A,B). Initially, Synapsin levels were very low and then rose rapidly, increasing 6-fold during the first decade of life to reach adult levels at ~9 years of age (Figure 2A; 3τ = 8.7 +/− 5.1 years; curve fit, *R* = 0.66; *p* < 0.0001). Analysis of the developmental stages showed a significant increase in Synapsin (Figure 2B; ANOVA, *p* < 0.0001). There was a significant increase in expression of Synapsin between Neonates (<0.3 years) and Older Children (5–11 years; Tukey’s, *p* < 0.01), that persisted through Teens (12–20 years; Tukey’s, *p* < 0.001), Young Adults (21–55 years; Tukey’s, *p* < 0.0001), and Older Adults (55+ years; Tukey’s, *p* < 0.05). We also found a significant increase in Synapsin expression between Infants (0.3–1 Year) and Older Children (5–11 years; Tukey’s, *p* < 0.05), Teens (12–20 years; Tukey’s, *p* < 0.05), and Young Adults (21–55 years; Tukey’s, *p* < 0.01).

Synaptophysin expression during early development was more variable and higher than Synapsin. We did not find a significant curve-fit to Synaptophysin expression and chose to use a simple weighted average to plot a curve describing Synaptophysin expression across the lifespan (Figure 2C). Also, there were no significant differences in Synaptophysin among the binned developmental age groups (Figure 2D; ANOVA, *p* = 0.09), suggesting that Synaptophysin is relatively constant in human visual cortex across the lifespan. These results point to inter-individual variability in Synaptophysin expression at the younger ages (<5 years) which may mask early developmental changes.

### Post-synaptic scaffolding proteins develop into late childhood

Both excitatory and inhibitory mechanisms are involved in developmental synaptic plasticity, and the physiological E-I balance is central to the initiation of the critical period in visual cortex. We examined post-synaptic development of glutamatergic (excitatory) and GABAergic (inhibitory) systems by quantifying the expression of 2 scaffolding proteins—PSD-95 and Gephyrin. PSD-95 anchors the excitatory GluA and GluN receptors, and is required for receptor function (Béïque et al., 2006). Gephyrin anchors the inhibitory GABA_A_ receptors, and is required for receptor stabilization (Yu et al., 2007). Together, the expression of PSD-95 and Gephyrin provides information about the development of excitatory and inhibitory synapses (Keith and El-Husseini, 2008) and the E-I balance that is crucial for ocular dominance plasticity in visual cortex (Hensch and Fagiolini, 2005).

**Figure 3:**
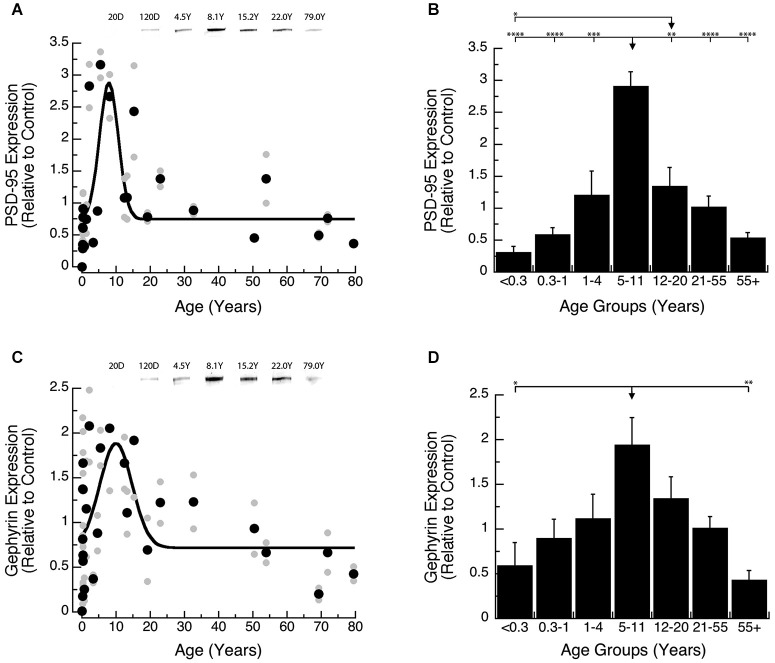
**Developmental changes in PSD-95 (A,B) and Gephyrin (C,D) expression in human visual cortex. (A,C)** Gray dots are results from all runs, and black dots are the average for each sample. Example bands are shown above the graphs. **(B,D)** Group means and standard error for each developmental stage are plotted. **(A)** A Gaussian function was fit to all the PSD-95 data points (*R* = 0.58; *p* < 0.0001), and a peak in expression was reached at 8 years of age (peak = 8.0 +/− 0.7 years). **(B)** There was a significant difference in expression of PSD-95 between the groups (ANOVA, *p* < 0.0001), and the statistical significance of the difference between pairs of development stages as determined by Tukey’s *post hoc* comparisons are plotted (*p < 0.05; **p < 0.01; ***p < 0.001; ****p < 0.0001). **(C)** A Gaussian function was fit to all the Gephyrin data points (*R* = 0.48; *p* < 0.0005), and a peak in expression was reached at 10.0 years of age. **(D)** There was a significant difference in expression of Gephyrin between the groups (ANOVA, *p* < 0.005), and the statistical significance of the difference between pairs of development stages as determined by Tukey’s *post hoc* comparisons are plotted (*p < 0.05; **p < 0.01; ***p < 0.001; ****p < 0.0001).

Development of PSD-95 and Gephyrin expression followed similar developmental trajectories, increasing into late childhood and then declining into adulthood. Development of PSD-95 increased ~10-fold to reach a peak at 8 years of age (Figure 3A; peak = 8.0 +/− 0.7 years; curve fit, *R* = 0.58; *p* < 0.0001), and then declined ~5-fold. Comparison of PSD-95 expression levels among developmental stages (Figure 3B; ANOVA, *p* < 0.0001) showed that it was significantly higher in Older Children (5–11 years) relative to all other age groups: Neonates (<0.3 years; Tukey’s, *p* < 0.0001), Infants (0.3–1 Year; Tukey’s, *p* < 0.0001), Young Children (1–4 years; Tukey’s, *p* < 0.001), Teens (12–20 years; Tukey’s, *p* < 0.01), Young Adults (21–55 years; Tukey’s, *p* < 0.0001), Older Adults (55+ years; Tukey’s, *p* < 0.0001). We also found that teens (12–21 years) had higher expression levels of PSD-95 than Neonates (<0.3 years; Tukey’s, *p* < 0.05).

Gephyrin expression increased ~3-fold and model-fitting of a peak function found the maximum expression at 10 years of age (Figure 3C; peak = 10.0 +/− 1.3 years; curve fit, *R* = 0.48; *p* < 0.0005), followed by a subsequent ~4-fold decline. We saw a similar developmental profile for Gephyrin when analyzing the age groups (Figure 3D; ANOVA, *p* < 0.005). Expression levels of Gephyrin were significantly higher in Older Children (5–11 years), than Neonates (<0.3 years; Tukey’s, *p* < 0.05), and Older Adults (55+ years; Tukey’s, *p* < 0.01).

### Waves of inter-individual variability across the lifespan

**Figure 4:**
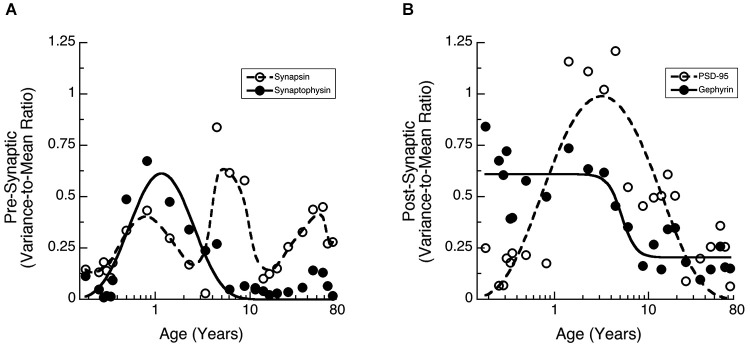
**Development of the Variance-to-Mean Ratio (VMR) for Synapsin and Synaptophysin (A), as well as PSD-95 and Gephyrin (B)**. **(A)** Synapsin (open circles, dashed line, weighted average function) had 3 peaks in VMR across the lifespan (1 year, 5–10 years, and older adults). Synaptophysin (filled dots, solid line; a * exp(b/x + c * x), *R* = 0.86, *p* < 0.0001) had a peak in VMR around 1 year of age. **(B)** PSD-95 (open circles, dashed line; a * exp(b/x + c * x), *R* = 0.82, *p* < 0.0001) had a peak in VMR throughout childhood. Gephyrin (filled dots, solid line; a + (b − a)/(1 + (x/c)^d^), *R* = 0.88, *p* < 0.0001) had a decline in VMR starting at about 5 years of age (inflection point = 5.2 years +/− 0.9).

Many studies of human brain development and function have found large inter-individual variations. We noticed greater inter-individual variability in expression of synaptic proteins in human cortex than we had found for rat cortex (Pinto et al., 2013). To quantify that inter-individual variability and determine if it changes across the lifespan we calculated the Fano factor (VMR) for a running window across 3 adjacent ages and then fit functions (Synaptophysin, PSD-95, Gephyrin) or a weighted average (Synapsin) to the VMRs to capture the pattern of change (Figure 4). The VMR for Synapsin and Synaptophysin showed waves of inter-individual variability across the lifespan (Figure 4A). Synaptophysin had a prominent peak in the VMR at 1 year of age and a smaller bump later in adulthood (curve fit, *R* = 0.86, *p* < 0.0001). Synapsin variability also had a peak at about 1 year, then a second peak during late childhood (5–10 years), and a third one during late adulthood. The post-synaptic proteins had different patterns of inter-individual variability (Figure 4B). Gephyrin VMR was high during early development up to about 5 years of age (inflection point = 5.2 years +/− 0.9), then declined through later childhood and remained low during adolescence and adulthood (curve fit, *R* = 0.88, *p* < 0.0001). In contrast, the VMR for PSD-95 expression was low in infants (<1 year) and adults (>20 years) but had a prominent peak during childhood and was elevated throughout adolescence (curve fit, *R* = 0.82, *p* < 0.0001). The waves of inter-individual variability for the synaptic proteins highlight developmental stages when there is greater variation in synaptic development and those may signify ages of vulnerability for specific aspects of synaptic maturation.

### Pre- and post-synaptic balances develop within first year in human visual cortex

Each pair of proteins support functioning of the pre-synaptic (Synapsin and Synaptophysin) or post-synaptic (PSD-95 and Gephyrin) terminal. On the pre-synaptic side, Synapsin and Synaptophysin are both required for pre-synaptic function (Hopf et al., 2002), and the balance between the two proteins affects the dynamics of vesicle cycling because Synapsin regulates exocytosis and Synaptophysin regulates endocytosis. On the post-synaptic side, PSD-95 and Gephyrin regulate the number of excitatory and inhibitory synapses (Prange et al., 2004; Lardi-Studler et al., 2007; Keith and El-Husseini, 2008) and together affect the physiological E-I balance. To quantify the balance between each pair of synaptic proteins, we calculated 2 indices; a Pre-Synaptic Index: (Synapsin−Synaptophysin)/(Synapsin+Synaptophysin), and a Post-Synaptic Index: (PSD-95−Gephyrin)/(PSD-95+Gephyrin). The indices range from +1 to −1, with positive values indicating relatively more Synapsin (Pre-Synaptic Index) or PSD-95 (Post-Synaptic Index), and negative values indicating relatively more Synaptophysin (Pre-Synaptic Index) or Gephyrin (Post-Synaptic Index).

**Figure 5:**
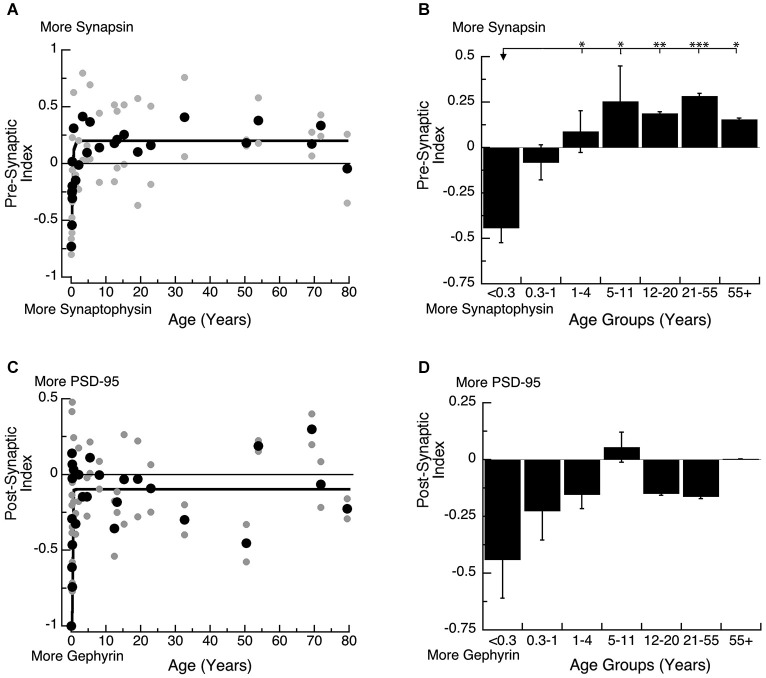
**Developmental changes in the pre-synaptic (A,B) and post-synaptic (C,D) index in human visual cortex. (A,C)** Gray dots are results from all runs, and black dots are the average for each sample. Example bands are shown above the graphs. **(B,D)** Group means and standard error for each developmental stage are plotted. **(A)** An exponential decay function was fit to all the pre-synaptic index data points (*R* = 0.67, *p* < 0.0001), and adult levels are defined as 3t (3*t* = 11.7 +/− 4.1 months). **(B)** There was a significant difference in expression of the pre-synaptic index between age groups (ANOVA, *p* < 0.0005) and the statistical significance of the difference between pairs of development stages as determined by Tukey’s *post hoc* comparisons are plotted (*p < 0.05; **p < 0.01; ***p < 0.001; ****p < 0.0001). **(C)** An exponential decay function was fit to all the post-synaptic index data points (*R* = 0.51, *p* < 0.0001), and adult levels were defined as 3t (3.5 +/− 1.8 months). **(D)** There were no significant differences in expression of the post-synaptic index among the developmental stages (ANOVA, *p* = 0.18).

Both the pre- and post-synaptic indices developed very rapidly in the first year (Figure 5). On the pre-synaptic side, there was an early switch from relatively more Synaptophysin expression to slightly more Synapsin expression, and mature-levels were reached by ~12 months of age (Figure 5A; 3τ = 11.7 +/− 4.1 months; curve fit, *R* = 0.67, *p* < 0.0001). We found a similar developmental profile with a significant switch in expression of the pre-synaptic index among the age groups (Figure 5B; ANOVA, *p* < 0.0005). Expression levels switched from more Synaptophysin in Neonates (<0.3 years) to more Synapsin in Younger Children (1–4 years; Tukey’s, *p* < 0.05) and that persisted through Older Adults (55+ years; Tukey’s, *p* < 0.05). This switch in the balance between Synapsin and Synaptophysin suggests that pre-synaptic function of vesicle endo- and exo-cytosis matures within the first year.

On the post-synaptic side, we found a rapid switch from much more Gephyrin at the youngest ages, to a balance between Gephyrin and PSD-95 by ~5 months of age (Figures 5C,D; 3τ = 5.3 +/− 1.8 Months; curve fit, *R* = 0.54, *p* < 0.0001). The developmental stages showed a similar profile, switching from more Gephyrin to a balance between the 2 proteins. The rapid switch in the first few months, however, led to greater variability during the youngest developmental stages (<1 year), so the comparison between the age groups could not capture the change (Figure 5D; ANOVA, *p* = 0.18). This switch in the post-synaptic index suggests a rapid change in the E-I balance that would be expected to trigger the start of the critical period for ocular dominance plasticity in human visual cortex by 5 months of age.

### Principal component analysis

The synaptic proteome is a complex functional system, so to address the multidimensional nature of the development of these four proteins, we used a data-driven approach and analyzed all of the protein expression with SVD (Beston et al., 2010; Pinto et al., 2013). This allowed us to quantify the underlying principal components that capture a significant amount of the variance in the data from human visual cortex. The SVD found that the first principal component (PCA 1) explained the greatest proportion of the variance (64%), the second (PCA 2) explained 20% of the variance and the combination of PCA 1 and PCA 2 accounted for >80% of the variance an amount typically used to identify nontrivial components (North et al., 1982). A Monte Carlo simulation showed that the first principal component accounted for a significant proportion of the variance (PCA 1, *p* < 0.0001), and the second a trend (PCA 2, *p* = 0.09), but PCA3 and PCA4 were not significant (*p* > 0.2). Based on these two rules PCA 1 and PCA 2 were identified as significant components and used for the subsequent analyses.

**Figure 6:**
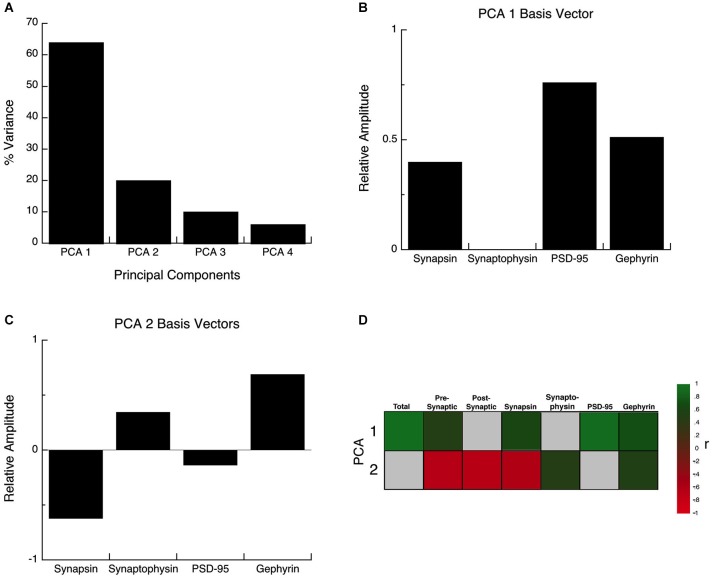
**Principal component analysis. (A)** The percent variance captured by each component of the SVD analysis of protein expression in human visual cortex. The first 2 principal components represent 84% of the SVD. **(B)** The influence of each protein on the first principal component was reflected by the relative amplitude in the basis vectors. **(C)** The influence of each protein on the second principal component was reflected by the relative amplitude in the basis vectors. **(D)** Significant correlations (colored cells) between the first 2 principal components and the combinations of proteins derived from the basis vectors. The color indicates the magnitude (represented by color intensity) and direction (green indicates positive, red indicates negative) of significant correlations (Bonferroni corrected, *p* < 0.0035).

The principal components represent a linear combination of the expression of the four proteins and the influence that each protein had on PCA1 or PCA2 was reflected in the relative amplitude of the basis vector (Figures 6B,C). Analyzing the basis vectors for PCA1 and PCA2 was an important, 2-step process, that we used to link the principal components with relevant biological mechanisms (Beston et al., 2010; Pinto et al., 2013). First, we computed the basis vectors; this provides insights regarding the biological mechanisms driving the data. The basis vectors for PCA 1 (Figure 6B) showed positive contributions from all four of the proteins (albeit a very small amount for Synaptophysin), indicating that PCA 1 is driven by the combined expression of the 4 proteins (synaptic protein expression). For PCA 2, the basis vectors showed opposite directions for the pre-synaptic markers (Synapsin and Synaptophysin), and opposite directions for the post-synaptic markers for excitatory and inhibitory synapses (PSD-95 and Gephyrin) (Figure 6C). The opposite directions for the pairs of proteins suggests that PCA 2 is linked with changes in the balance between the pre- and post-synaptic protein pairs.

For the second step, we generated a set of correlations between the two significant principal components (PCA 1 and PCA 2), and the four proteins, two indices, and a new measure identified in step one (synaptic protein expression). To account for multiple comparisons, we performed a Bonferroni correction, and then displayed significant correlations between the two principal components and the seven measures (Figure 6D; green and red squares, *p* < 0.0035). The pattern of correlations provides information that describes the biological links for each principal component. The first principal component had the greatest correlation with the sum of the 4 proteins (*R* = 0.9764, *p* < 0.0001), and was also correlated with Synapsin (*R* = 0.6882, *p* < 0.0001), PSD-95 (*R* = 0.9486, *p* < 0.0001), Gephyrin (*R* = 0.7651, *p* < 0.0001), and the pre-synaptic index (*R* = 0.5480, *p* < 0.0001). The second principal component was negatively correlated with changes in Synapsin (*R* = −0.5944, *p* < 0.0001), and positively correlated with Synaptophysin (*R* = 0.528, *p* < 0.001) and Gephyrin (*R* = 0.5717, *p* < 0.0001) expression, and had slightly stronger negative correlations with the balances for the pre-synaptic (*R* = −0.6105, *p* < 0.0001), and post-synaptic (E-I) (*R* = −0.625, *p* < 0.0001) indices.

**Figure 7:**
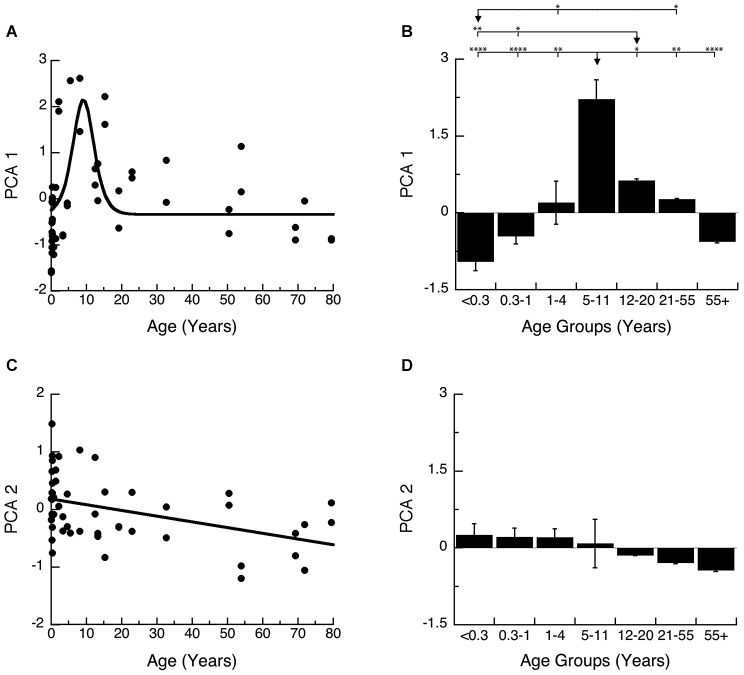
**Developmental changes in the principal components 1 and 2 in human visual cortex. (A)** Principal component 1. A logistics function was fit to the data. Principal component 1 had a peak in expression at 9 years of age (Figure 6; Peak = 9.2 +/− 0.7 years; curve-fit, *R* = 0.52, *p* < 0.0001). **(B)** Group mean and standard error for each developmental stage are plotted and the statistical significance (ANOVA, *p* < 0.0001) of the difference between pairs of development stages as determined by Tukey’s *post hoc* comparisons are plotted (*p < 0.05; **p < 0.01; ***p < 0.001; ****p < 0.0001). **(C)** Principal component 2. A linear function was fit to the data (*R* = 0.43, *p* < 0.005). **(D)** Group mean and standard error for each developmental stage are plotted and there were no significant differences in expression among experimental groups (ANOVA, *p* = 0.11).

We plotted PCA 1 (Figures 7A,B) and PCA 2 (Figures 7C,D) as a function of age, and the binned developmental age groups. The first principal component was strongly correlated with the sum of the 4 proteins (*R* = 0.9764, *p* < 0.0001) and was well fit by a logistic function with peak expression at ~9 years of age (Figure 7A; Peak = 9.2 +/− 0.7 years; curve-fit, *R* = 0.52, *p* < 0.0001) (Figures 7A,B). We found a similar developmental profile when comparing the age groups (Figure 7B). There were significant differences in expression among the developmental stages (ANOVA, *p* < 0.0001), with Older Children (5–11 years) having significantly higher PCA 1 than all other age groups. Teens (12–20) also had relative higher PCA 1 when compare with Neonates (<0.3 years; Tukey’s, *p* < 0.01), and Infants (0.3–1 Year; Tukey’s, *p* < 0.05), while Neonates (<0.3 years) had relatively less when compare with Young Children (1–4 years; Tukey’s, *p* < 0.05), and Young Adults (21–55 years; Tukey’s, *p* < 0.05). Together, these results show a prolonged developmental increase in synaptic protein expression that continues into older children, and suggests a long period of synaptic stabilization in human visual cortex.

The second principal component was most strongly correlated with the pre-synaptic (*R* = −0.6105, *p* < 0.0001) and post-synaptic indices (E-I balance; *R* = −0.625, *p* < 0.0001) and therefore is related to developmental progression and maturation. Development of PCA 2 was well fit by a linear function (Figure 7C; *R* = 0.43, *p* < 0.005) and showed a very long and shallow decline. When comparing the developmental stages, the magnitude of change in PCA 2 is small and there was an overall trend for a decline across age groups (Figure 7D; ANOVA, *p* = 0.11).

### Comparing human and rat synaptic protein expression

**Figure 8:**
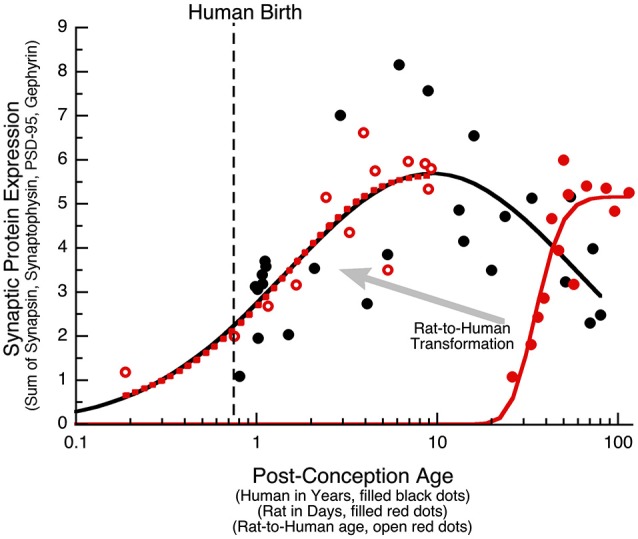
**Transformation of rat data to human age**. The sum of the four synaptic proteins (synaptic protein expression) is plotted for human (black dots, age in years), rat (red dots, age in days), and the rat results transformed into human age (open red dots). Human data was fit with a Gaussian function (*R* = 0.74, *p* < 0.0001), and reached a peak in expression at 9 years of age (Peak: x-axis = 9.4 years, y-axis = 5.7). Rat data was fit with a decay function (*R* = 0.92, *p* < 0.0001), and reached maximum expression at P55 (Peak: x-axis = 54.5 days, y-axis = 5.2). The human and rat alignment was done by normalizing to the peak expression (Human Expression at 9 Years/Rat Expression at 55 Days), then determining the offset on the x-axis to align the rat curve (dotted red curve) with the human curve (solid black curve) of synaptic protein expression, and then applying that transformation to each of the rat data points. The transformed rat data was plotted (open red dots, dotted red curve) in human equivalent units. A large portion of the human (black) and transformed rat (dotted red) curves approximated a linear increase, allowing for a simple alignment of rats age in days with human post-conception age in years (Rat Age (Days) = 11 + 5.5 * Human Post Conception Age (Years)).

The PCA identified the sum of the 4 proteins as the main source of variance for development of human visual cortex. In another study, we quantified expression of the same synaptic proteins (Synapsin, Synaptophysin, PSD-95, and Gephyrin) in developing rat cortex, and using PCA analysis found that the largest portion of the variance was also accounted for by the sum of the four proteins (Pinto et al., 2013). Together, these data sets provide a unique opportunity to run a parallel comparison between human and rat cortical development, and to determine how to translate synaptic age between these species. First, we quantified the sum of the synaptic proteins in human visual cortex and plotted that as a function of post-conception age (Figure 8, black dots and curve). In human visual cortex, development of synaptic protein expression (black dots) followed a trajectory that was well fit by a Gaussian function with the peak of expression at ~9 years of age (Figure 8; curve-fit, *R* = 0.7376, *p* < 0.0001). Second, we plotted the development of synaptic protein expression in rat visual cortex (Pinto et al., 2013; Figure 8) and transformed rat ages (P4–P93) to post-conception days. In rat visual cortex (open red dots), development of synaptic protein expression for the range of ages followed a decay function that reached a maximum expression at 55 days post-conception (Figure 8; curve-fit, *R* = 0.9215, *p* < 0.0001).

The process of translating from rat to human expression involved determining the transformation required to shift the rat curve (solid red curve) along the x- and y-axes so that it matched the human curve and then applying that transformation to the rat data to visualize each point in corresponding human age. To normalize the maximum expression levels, we multiplied the rat data by the ratio of the maxima from the human and rat curves (Human Expression at 9 Years/Rat Expression at 55 Days). To shift the rat along the x-axis, we found the horizontal transformation necessary to align the rat curve (dotted red curve) with the human curve (solid black curve) for the sum of the synaptic proteins, and then applied that transformation to each of the rat data points. The transformed rat data were plotted (filled red circles, dotted red curve) in human equivalent units. This transformation showed similar developmental trajectories for synaptic proteins in rats aged P0 to P93 and humans aged 20 days to 8.5 years. A large portion of those curves approximated a linear increase, which facilitated a simple alignment between rat age in days and human post conception age in years (Rat Age (Days) = 11 + 5.5 * Human Post Conception Age (years)). Using this equation, we made a table (Table 2) to illustrate corresponding ages when protein expression was comparable, and highlight known milestones in visual system plasticity in the two species. Interestingly, there was comparable expression at human birth and rat eye opening, suggesting that synaptic protein expression is driven by the initial strong visual stimuli experienced in both species. In addition, there was also comparable expression at the ages linked to the end of susceptibility for developing amblyopia in humans (~6 years) (Epelbaum et al., 1993; Keech and Kutschke, 1995) and the end of the critical period for ocular dominance plasticity in rats (P45) (Fagiolini et al., 1994). Together, these results show a simple translation between rat and human synaptic development in visual cortex, and suggest that the sum of this set of 4 synaptic proteins in human and rat visual cortex reflect comparable stages of visual system development.

**Table 2:** **Alignment of age between humans and rats with milestones for visual system developmental plasticity**.

Human visual milestones	Human age (years)	Rat age (days)	Rat visual milestones
	−0.6	4	
Birth	0.0	11	Eye opening
Start of the sensitive period for binocular vision (Banks et al., 1975)	0.4	14	Pre-critical period (Fagiolini et al., 1994; Feller and Scanziani, 2005)
Peak of the sensitive period for binocular vision (Banks et al., 1975)	0.9	17	
	1.7	21	Start of the critical period for ocular dominance plasticity (Fagiolini et al., 1994)
	2.5	25	
	3.2	28	Peak of the critical period (Fagiolini et al., 1994)
	3.8	31	
	4.6	35	
End of susceptibility for developing amblyopia (Epelbaum et al., 1993; Keech and Kutschke, 1995; Lewis and Maurer, 2005)	6.2	45	End of the critical period (Fagiolini et al., 1994)
	7.8	64	
	8.2	74	
	8.5	93	

## Discussion

The results of this study highlight the complex nature of synaptic development in human visual cortex, showing that there are both prolonged and rapid changes in synaptic proteins, plus waves of inter-individual variability. A simple transformation, however, provides a robust alignment of synaptic age between rats and humans. We have drawn three main conclusions from the results.

### Prolonged development of synaptic proteins in human primary visual cortex

First, there is prolonged development of pre- and post-synaptic proteins in human V1, suggesting that synaptic development and stabilization extends well into the childhood years. This prolonged development is similar to the pattern of development found in our previous studies of receptor subunit maturation during childhood (Murphy et al., 2005) and GABAergic protein changes across the lifespan (Pinto et al., 2010). Pre-synaptically, Synaptophysin expression was relatively constant across the lifespan, while Synapsin expression increased until about 6 years of age. Both Synapsin and Synaptophysin are required for stabilization of pre-synaptic boutons (Hopf et al., 2002) and perhaps the increase in Synapsin expression during childhood promotes pre-synaptic stabilization. Post-synaptically, both PSD-95 and Gephyrin expression increased during childhood and then declined through adulthood. PSD-95 anchors glutametergic receptors affecting the strength of excitatory glutamatergic synapses (Schnell et al., 2002; Colledge et al., 2003; Stein et al., 2003; Ehrlich and Malinow, 2004), and nearby post-synaptic densities compete for the available pool of PSD-95 (Gray et al., 2006). These roles for PSD-95 point to prolonged maturation of excitatory synapse functioning in human visual cortex and then age-related weakening of glutamatergic synapses. Previously, we found a similarly slow development of glutamate receptor subunit expression in human visual cortex (Murphy et al., 2005) and the age-related decline in PSD-95 suggests a concomitant loss of glutamate receptors. An increase in Gephyrin cluster density reduces motility of the protein among neighboring inhibitory (GABAergic) synapses (Kuriu et al., 2012). Thus, the developmental increase in Gephyrin expression probably reflects prolonged maturation and stabilization of inhibitory synapses, followed by less stability in the aging human visual cortex. These prolonged changes in Gephyrin are very similar to the results of our previous study of GABAergic proteins in human visual cortex (Pinto et al., 2010). The prolonged developmental trajectories for the four synaptic proteins suggests a slow process of synaptic stabilization in human visual cortex. Interestingly, this prolonged timing coincides with clinical reports highlighting an extended period of susceptibility for developing amblyopia in children (Epelbaum et al., 1993; Keech and Kutschke, 1995).

Early studies of synaptic development in human cortex using electron microscopy (EM) found a peak in the number of synapses in V1 at about 8–11 months of age (Huttenlocher et al., 1982). In contrast, we found that maturation of synaptic proteins continues for years, until later childhood (about 8–10 years of age). The EM studies defined a synapse as the presence of synaptic vesicles, as well as a pre- and post-synaptic density. Perhaps the early development of pre- and post-synaptic balances found in the current study reflect that initial presence of a full synaptic complement. Counts of dendritic spines also show an early rise with adult levels reached at 2 years of age (Michel and Garey, 1984), which is similar to the current findings for maturation of the E-I balance. Using expression of synaptic proteins is a different measure of synaptic development than the anatomical techniques and this may contribute to different conclusions about the pace of development. Our previous study of rat visual cortex development (Pinto et al., 2013), however, found a close match between the developmental trajectories for these synaptic proteins and EM counts of synapses in development rat cortex (Blue and Parnavelas, 1983). In fact, even the brief peak in the counts of inhibitory synapses aligned with a brief bump in Gephyrin expression. EM studies are very labor intensive, which limits them to using small numbers of cases to count synapses. There is no doubt that the waves of synaptic inter-individual variability found in this study could affect the measurement of developmental trajectories, especially when a small number of cases are sampled at ages with high variation. Finally, new ultra high resolution neuroanatomical techniques, such as array tomography, are visualizing synapses with exquisite detail by combining the best of the classical neuroanatomy techniques, with labeling of synaptic proteins (e.g., Synapsin, Synapstophysin, PSD-95, Gephyrin) and computer 3D reconstruction (Micheva and Bruchez, 2012). These new studies have found a much higher density of synapses in human cortex than the old EM work (Micheva et al., 2010) and are revealing a diversity of synapses that is important for measuring neural circuit development, structure, and function (O’Rourke et al., 2012). It will be exciting for future studies of human cortical development to combine the current results about synaptic protein development with ultra high resolution neuroanatomy to see maturation of the human synaptic connectome.

### Early switch in balance of pre- and post-synaptic proteins

Second, within the first year of life, we found abrupt switches in both pre- and post-synaptic protein balances. This contrasts with the gradual development of individual proteins found in this and our previous studies of human visual cortex (Murphy et al., 2005; Pinto et al., 2010; Williams et al., 2010) and suggests that subtle changes in the relative amounts of synaptic proteins may affect functional maturation of synapses. Since the pre-synaptic proteins, Synapsin and Synaptophysin regulate different aspects of vesicle cycling (endocytosis and exocytosis, respectively), the balance will affect the probability of transmitter release, especially with strong or sustained patterns of visually-driven activity. The rapid pre-synaptic development likely affects the maturation of neural signal-to-noise and contributes to the change from weak and sluggish to strong and sustained firing patterns. That change is necessary for both efficient synaptic transmission (Rust et al., 2002) and driving neuroplasticity mechanisms needed for experience-dependent development of receptive field properties (Smith and Trachtenberg, 2007).

The post-synaptic balance changed even faster, occurring at about 5 months of age. The balance switched from much more Gephyrin to roughly equal expression of Gephyrin and PSD-95. This rapid switch of the E-I balance in human visual cortex parallels the change we found for rat visual cortex at the start of the critical period for ocular dominance plasticity (Pinto et al., 2013) and provides an indication of the balance between excitatory and inhibitory synapses that contribute to the physiological E-I balance (Prange et al., 2004; Lardi-Studler et al., 2007; Keith and El-Husseini, 2008). Perhaps the rapid switch at 5 months of age in the relative amount of Gephyrin and PSD-95 contributes to triggering a period of heightened ocular dominance plasticity in human visual cortex.

Early in development, animals studies have shown that the physiological E-I balance favors excitation and a strong increase in inhibition is known to trigger the onset of the critical period for ocular dominance plasticity (Fagiolini and Hensch, 2000; Maffei and Turrigiano, 2008). Our finding of more Gephyrin during early development may seem counter intuitive to those physiological findings, but initially GABA_A_ receptors are depolarizing due to an abundance of the immature chloride co-transporters NKCC1. During development, there is a change from NKCC1 to KCC2, which turns GABA_A_ receptors hyperpolarizing (Ben-Ari, 2002). During human cortical development that change takes place in the first year (Dzhala et al., 2005) and by 3–5 months of the age there is a strong increase in inhibition that could trigger the onset of the critical period.

Functional aspects of binocular vision emerge at 3–5 months of age (Braddick et al., 1980, 1983; Held et al., 1980), and maturation of cortical binocularity in infants is driven by visual experience (Jandó et al., 2012). Indeed, the original description of the sensitive period for development of binocularity in children identified it as starting several months after birth (range 4–6 months) and peaking at 1–3 years of age (Banks et al., 1975). The timing for the start of the sensitive period for binocular vision coincides with the age when we found an abrupt switch from more Gephyrin to more PSD-95. That post-synaptic switch is suggestive of a rapid change in the physiological E-I balance that could trigger the start of the critical period for ocular dominance plasticity in human visual cortex. Furthermore, the similarity between the timing of the start of the sensitive period for binocular vision and the post-synaptic switch raises the intriguing possibility that maturation of human visual cortex has an early pre-critical period (before 3–5 months), similar to animal models (Feller and Scanziani, 2005), when binocular vision drives map refinement (Smith and Trachtenberg, 2007), and then a switch to a period of heightened binocular competition that drives ocular dominance plasticity. None of the synaptic markers that we measured had peaks of expression at 1–3 years of age when development of binocular vision is most sensitive to abnormal vision (Banks et al., 1975). But all of the markers had high inter-individual variability during that important stage for development of binocular vision. Since animal models have identified plasticity differences between pre-critical vs. critical period (Feller and Scanziani, 2005), it will be important to determine the timing of those stages in human cortex to understand experience-dependent development of human vision and facilitate translation of neuroplasticity-based treatments for amblyopia in children.

### Alignment of synaptic age for human and rat

Third, a simple transformation provides robust alignment of synaptic age between rat and human visual cortex. Using an informatics approach, we analyzed expression of all four synaptic proteins and found that the sum of the synaptic proteins (synaptic protein expression) accounts for most (64%) of the variance in development of human visual cortex across the lifespan. The sum of Synapsin, Synatophysin, PSD-95, and Gephyrin had a prolonged developmental trajectory that increased until late childhood and then decreased into aging. Recently, we applied the same analysis to rat visual cortex and found that expression of the same 4 proteins accounted for a surprisingly similar amount (64%) of the developmental changes rats (Pinto et al., 2013). This similarity between development of rat and human visual cortex provided us with a measure—synaptic protein expression—to use for determining the transformation to align rat and human synaptic age. A simple linear transformation showed good alignment between biologically relevant stages during development of rat and human visual system. Interestingly, the transformation also aligned a number of important milestones in visual system development and plasticity between rats and humans (Table 2). For example, synaptic protein expression levels were similar at birth in humans and eye opening (~P11) in rats. This is consistent with previous studies suggesting that rat cerebral cortex at P12–13 is comparable to human cerebral cortex at birth (Romijn et al., 1991), and that the initial strong visual stimulation experienced in both species drives similar increases in synaptic protein expression. In addition, synaptic protein expression at 6 years of age in humans lined up with 45 days of age in rats. These two ages coincide with the end of susceptibility for developing amblyopia in children (Epelbaum et al., 1993; Keech and Kutschke, 1995; Lewis and Maurer, 2005) and the end of the critical period for ocular dominance plasticity in rats (Fagiolini et al., 1994). These results suggest that developmental trajectories for synaptic protein expression reflect similar changes in experience-dependent neuroplasticity in both species and that the sum of expression for this small set of synaptic proteins is a good measure for aligning cortical age between species. In the future, this alignment may be useful for optimal translation from animal models of neuroplasticity to human developmental stages.

Although we have found a good translation for synaptic age of visual cortex between rats and humans, a number of challenges remain for converting other rodent models to humans. Even though rodents and humans share 75% 1:1 gene orthologs (Church et al., 2009), slight differences in the genome make translation difficult (Geerts, 2009). For example, mice lack the ApoE4 gene, which is a major risk factor for Alzheimer’s (Loring et al., 1996). These differences can render animal models incomplete and difficult for generating therapeutically relevant conclusions about human disease.

Our simple approach of quantifying a small set of synaptic proteins is a rapid, reliable, and relatively inexpensive way to characterize one aspect of cortical development. It will be important for future studies to examine maturation of synapses in different cortical layers and cell types, especially excitatory vs. inhibitory neurons, and to localize expression of synaptic proteins in various subcellular compartments. Answering those questions will depend on using modern methods that combine proteomics and neuroanatomical techniques to visualize the connectome (e.g., array tomography, (Micheva and Bruchez, 2012)). But both approaches, Western blotting and modern neuroanatomy, need to select appropriate synaptic markers from the hundreds of pre-synaptic proteins (Bayés and Grant, 2009) thousands of post-syanptic proteins (Collins et al., 2006; Trinidad et al., 2008). Furthermore, synaptic proteins are largely but not completely confined to the synapse and their expression is dynamically regulated leading to different amounts at each synapse. For example, even a simple distinction such as glutamatergic vs. GABAergic synapses can be complicated because the post-synaptic scaffolding proteins, PSD-95 and Gephyrin, are distributed across synaptic and extrasynaptic compartments (Aoki et al., 2001; Christie et al., 2002; Yoshii et al., 2003; Tyagarajan and Fritschy, 2014). In addition, the most common pre-synaptic markers, Synapsin and Synaptophysin, reflect the pool of vesicles rather than a specific pre-synaptic compartment. Together, these highlight the challenge of finding good markers for synapses. We are only beginning to reveal the complexity of the synaptic proteome (Emes and Grant, 2012) and need to be cautious when linking developmental or experience-dependent changes in synaptic proteins with the number of synapses or changes in physiological and behavioral properties.

The synaptic age transformation that we determined here can be used to help select better target ages for translating neuroplasticity therapies developed in various animal models. For example, recent advances have shown that application of neuroplasticity interventions (e.g., dark rearing, fluoxetine, environmental enrichment, food restriction) in adult rats promotes recovery from abnormal visual experience (He et al., 2007; Maya Vetencourt et al., 2008; Baroncelli et al., 2010; Spolidoro et al., 2011). Those studies describe P100 rats as “adults” due to their early sexual maturity (Quinn, 2005), but according to the current age alignment, the synaptic age of a P100 rat is more similar to an 8–10 year old child. The alignment of P100 rats with 8–10 year old children suggests that a P100 rat is probably more like a similarly aged mouse that still has significant ocular dominance plasticity (Tagawa et al., 2005; Lehmann and Löwel, 2008). Thus, older rats are probably needed for developing neuroplasticity treatments that translate well to human adolescents or adults.

A growing number of studies are finding complex patterns of cortical development that span the entire human lifespan. This is particularly apparent for expression of synaptic proteins (Murphy et al., 2005; Pinto et al., 2010; Williams et al., 2010) and the genes that encode them (Duncan et al., 2010). A full characterization of the changing synaptic proteome in human cortex is an important goal for identifying stages of human cortical development when neuroplasticity driven by disease may cause maladaptive change, and likewise when neuroplasticity driven by new treatments may promote adaptive change. The methods presented in this paper provide a simple protocol to measure 4 highly conserved synaptic proteins with techniques readily available in most labs and that can be easily applied to align synaptic age among other model species. Large-scale synaptic proteome studies using sensitive mass spectrometry, however, will undoubtedly uncover new information about species-specific levels of protein expression during synaptic development (Bayés et al., 2012).

The current results identify a new feature of human cortical development, the waves of inter-individual variability during childhood, which is an important insight for designing future studies of the developing human connectome. Finally, there is a clear need for additional parallel studies of neuroplasticity using animal models and humans. These will be key for understanding developmental plasticity in human cortex and extending that knowledge to identify and translate age-appropriate neuroplasticity therapies into optimal treatments for human diseases.

## Conflict of interest statement

The authors declare that the research was conducted in the absence of any commercial or financial relationships that could be construed as a potential conflict of interest.
